# Supplementary material for: Macrocyclic Ionic Liquids with Amino Acid Residues: Synthesis and Influence of Thiacalix[4]arene Conformation on Thermal Stability
Source: Molecules. 2022 Nov 18;27(22):8006. doi: 10.3390/molecules27228006 (PMC9698724; doi:10.3390/molecules27228006)
Supplement: Supplementary file 1 [file molecules-27-08006-s001.zip › molecules-2037692 Supplementary R2.pdf]

molecules

Supplementary Information

For

Macrocyclic Ionic Liquids with Amino Acid Residues: Synthesis and Influence of Thiacalix[4]arene Conformation on Thermal Stability

Olga Terenteva, Azamat Bikmukhametov, Alexander Gerasimov, Pavel Padnya and Ivan Stoikov

Content

Figures S1-S10. ^{1}H NMR spectra of the compounds **4, 6, 11-13, 15, 17-19, 21** . . . 2-6

Figures S11-S18. ^{13}C NMR spectra of the compounds **4, 6, 11-13, 15, 17-19, 21** . . . 7-10

Figures S19-S26. FT-IR spectra of the compounds **4, 6, 11-13, 15, 17-19, 21** . . . 11-14

Figures S27-S33. HRMS spectra of the compounds **4, 6, 11-13, 15, 17-19, 21** . . . 15-18

Figure S34. DSC curves of the compounds **16–21** 19

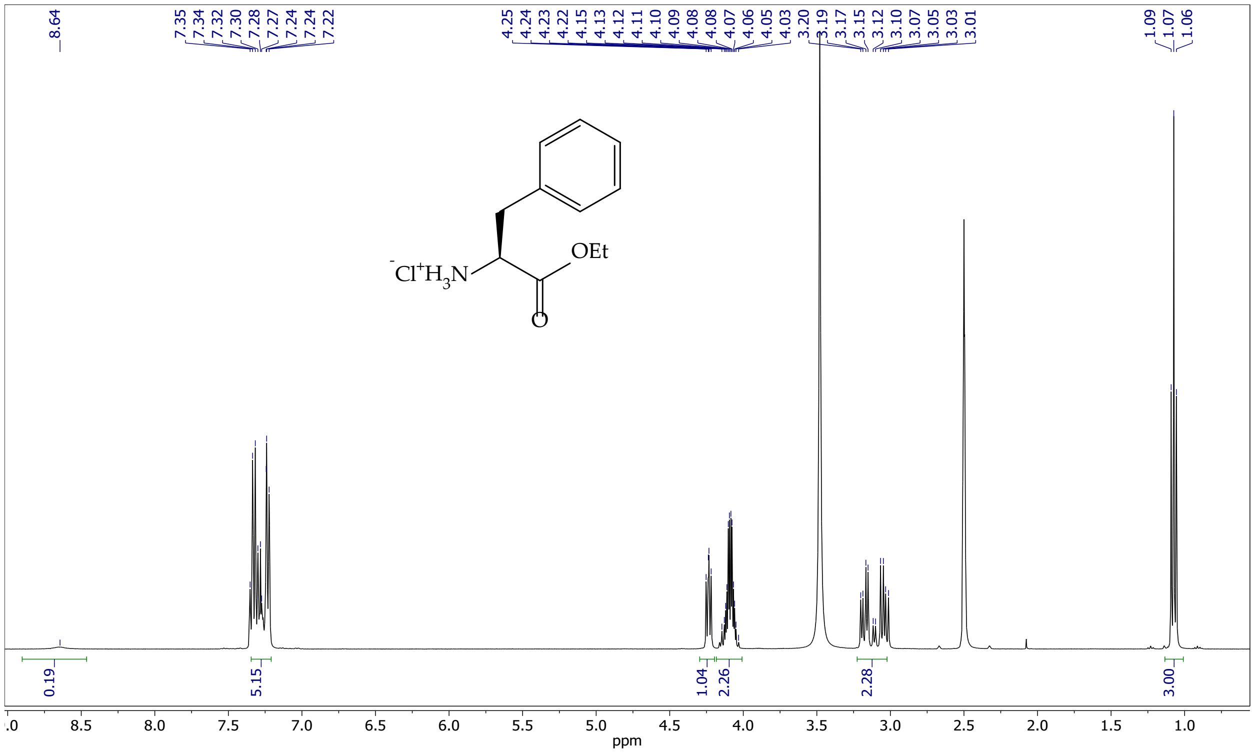

Figure S1. ^{1}H NMR spectrum of the compound **4**, DMSO-d_6, 298 K, 400 MHz.

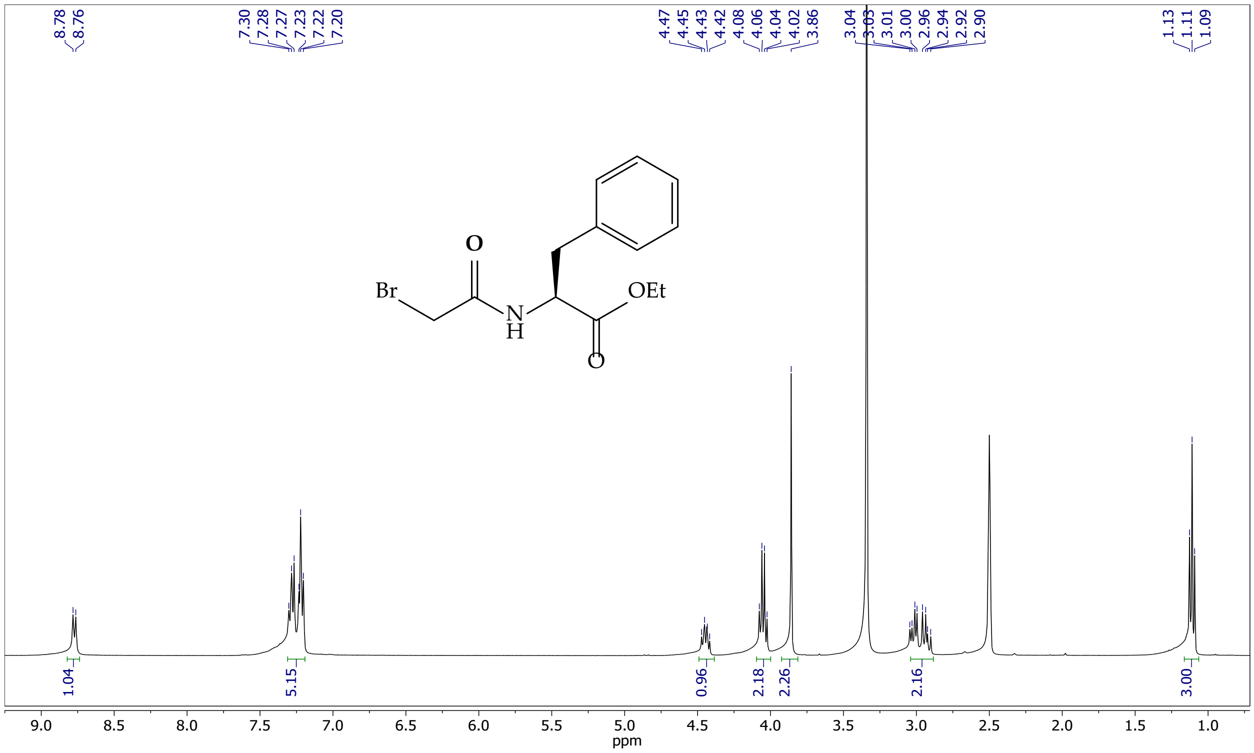

Figure S2. ^{1}H NMR spectrum of the compound **6**, DMSO-d_6, 298 K, 400 MHz.

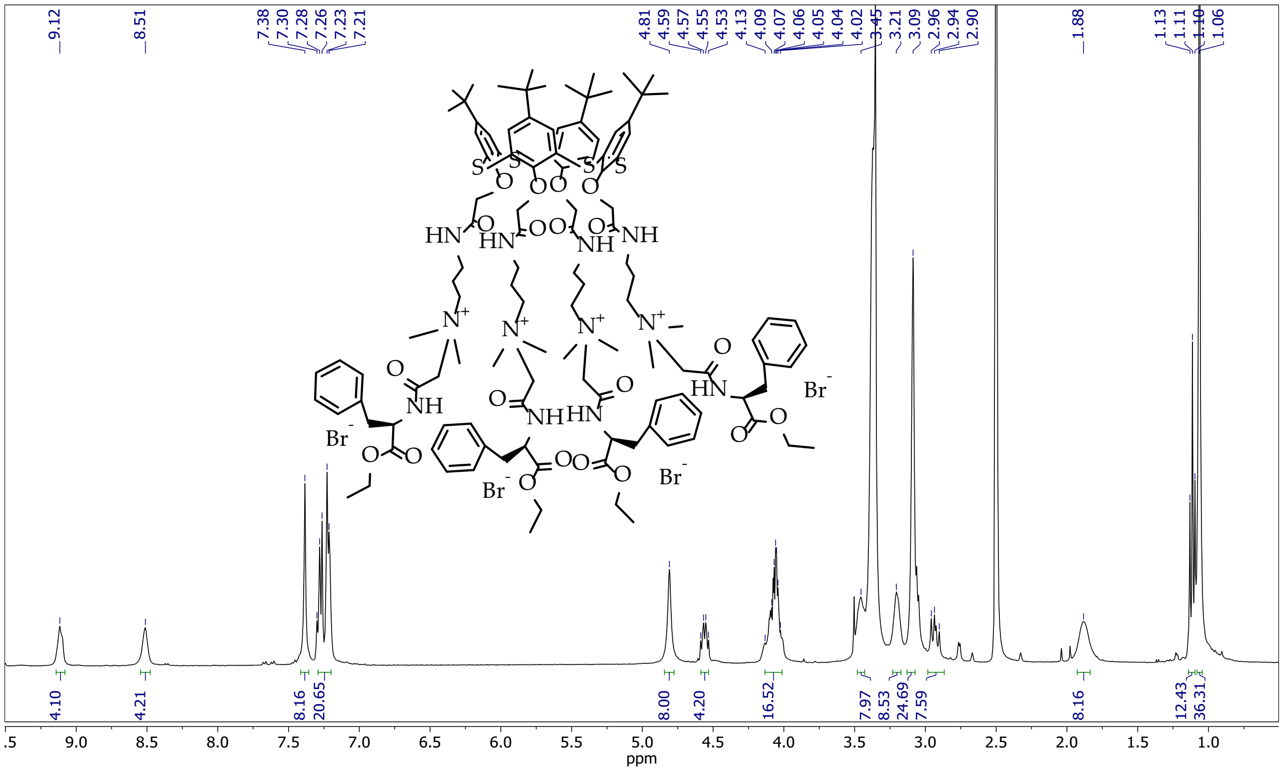

Figure S3. ^{1}H NMR spectrum of the compound **11** (*cone*), DMSO-d6, 298 K, 400 MHz.

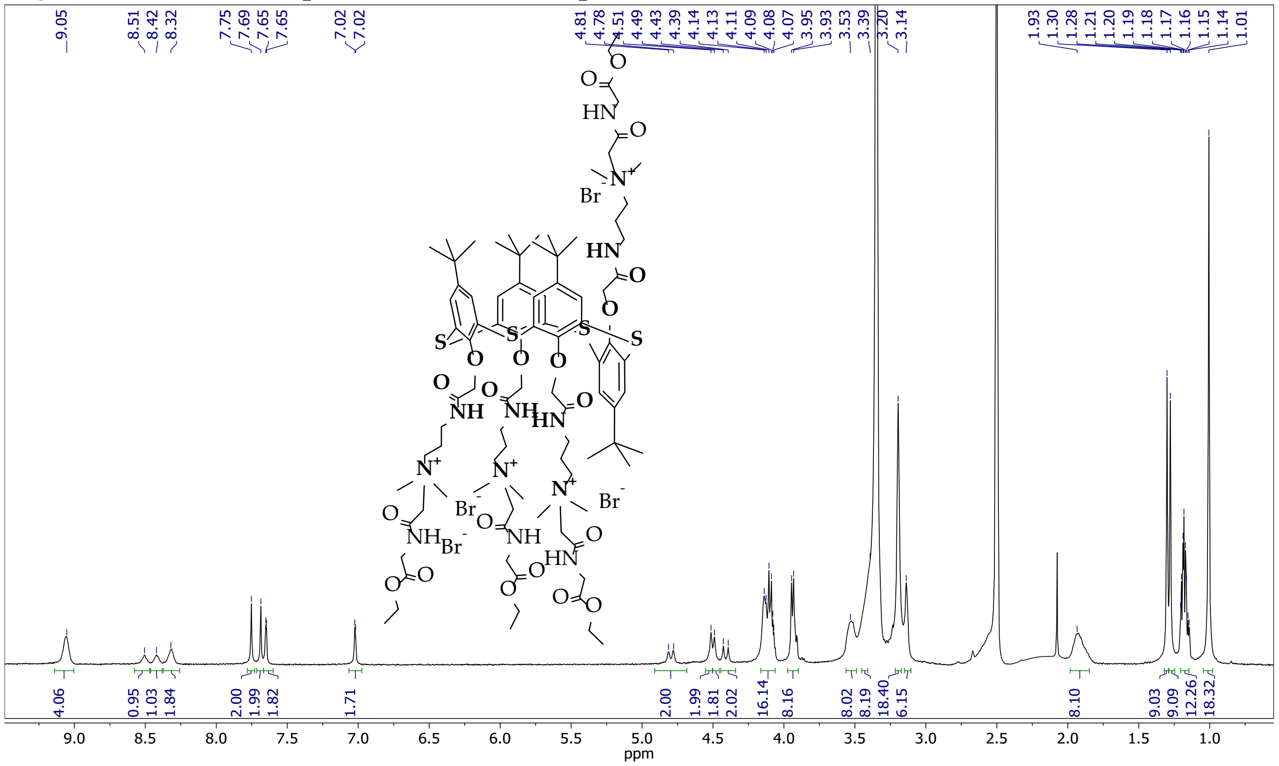

Figure S4. ^{1}H NMR spectrum of the compound **12** (*partial cone*), DMSO-*d*6, 298 K, 400 MHz.

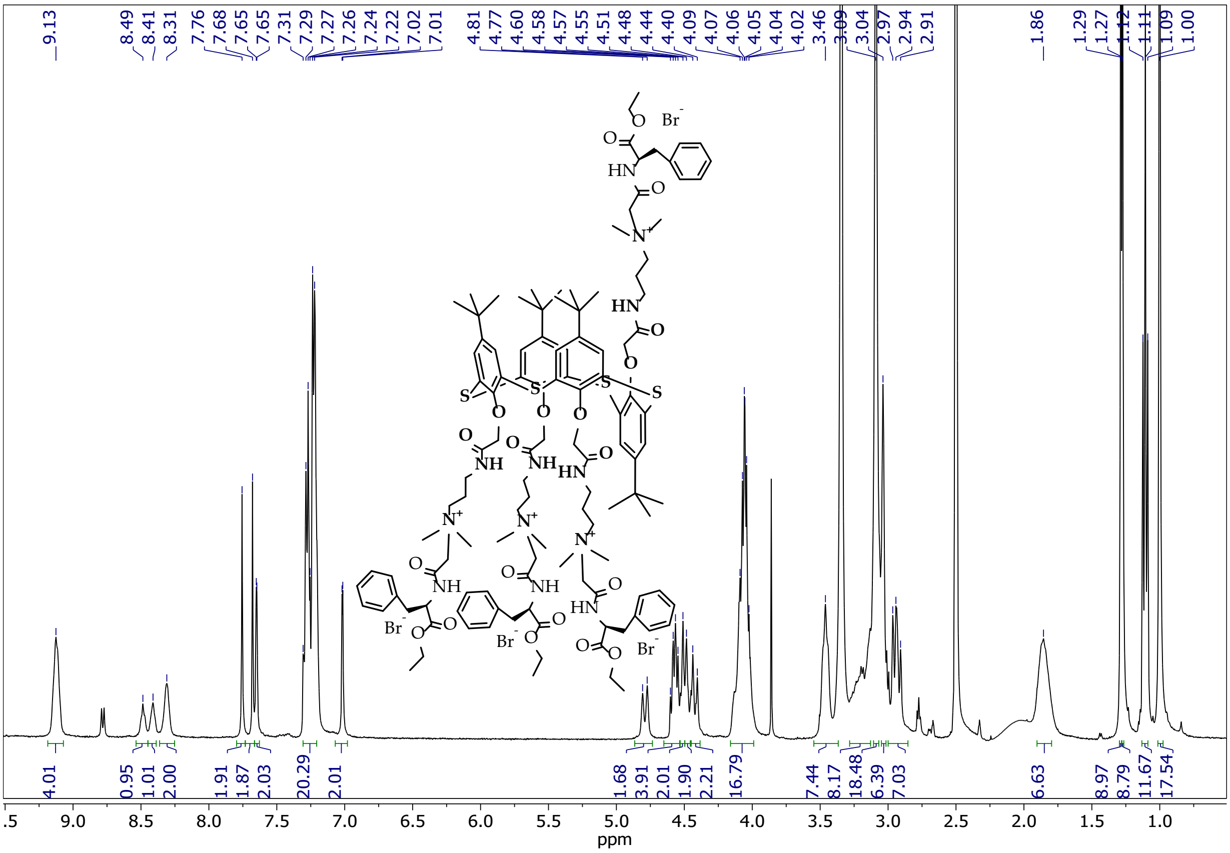

Figure S5. ^{1}H NMR spectrum of the compound **13** (*partial cone*), DMSO-d_6, 298 K, 400 MHz.

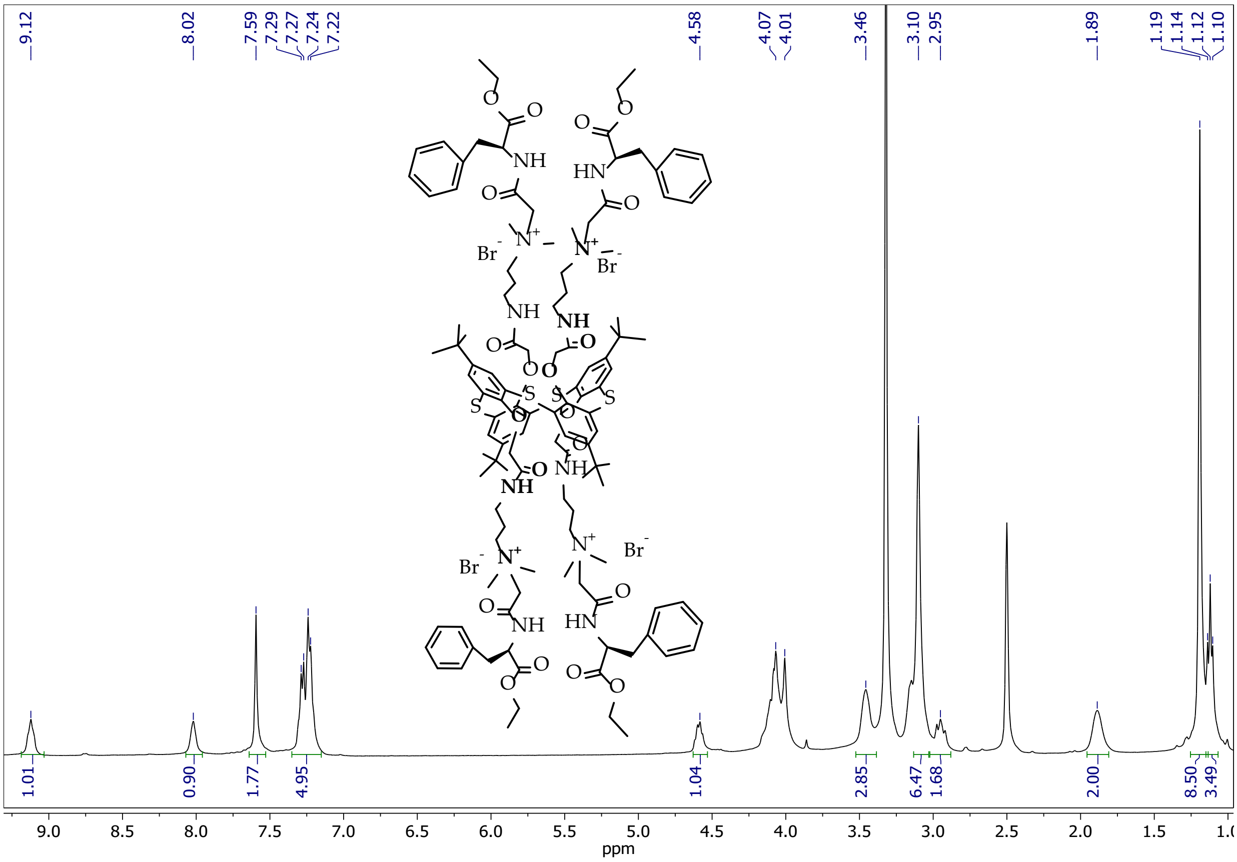

Figure S6. ^{1}H NMR spectrum of the compound **15** (*1,3-alternate*), DMSO-d_6, 298 K, 400 MHz.

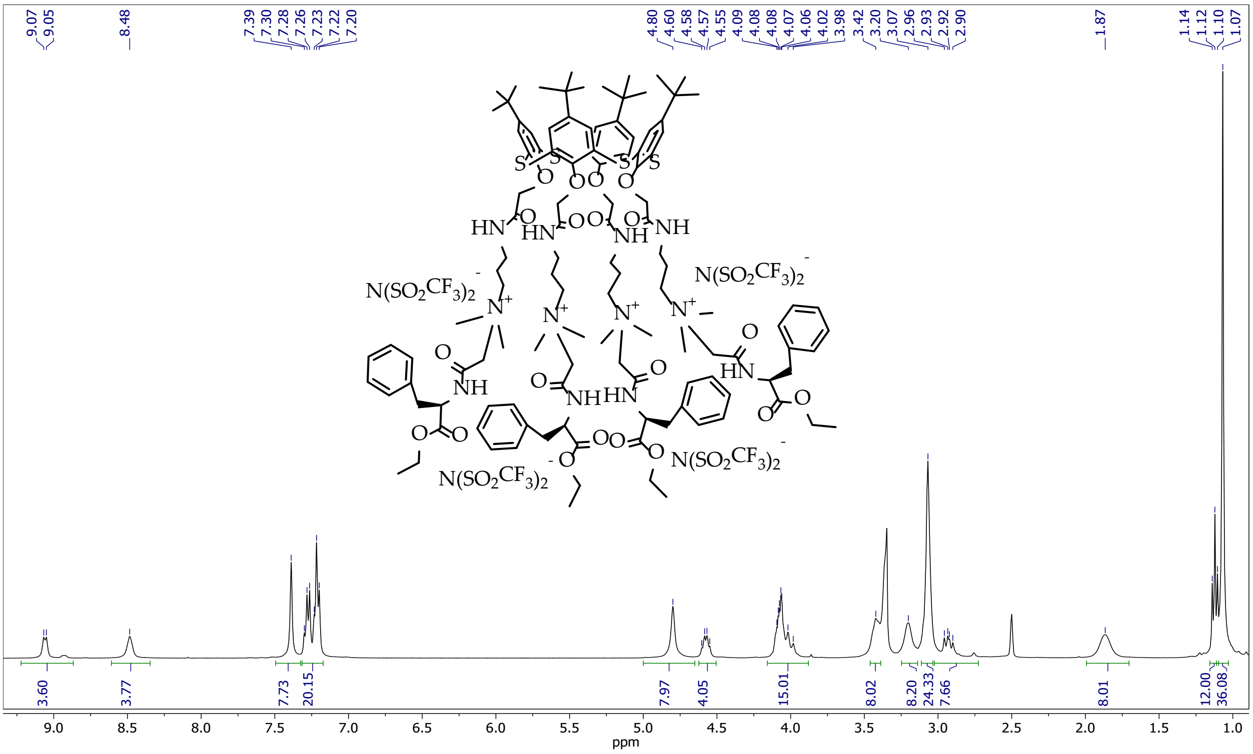

Figure S7. ^{1}H NMR spectrum of the compound **17** (*cone*), DMSO-d_6, 298 K, 400 MHz.

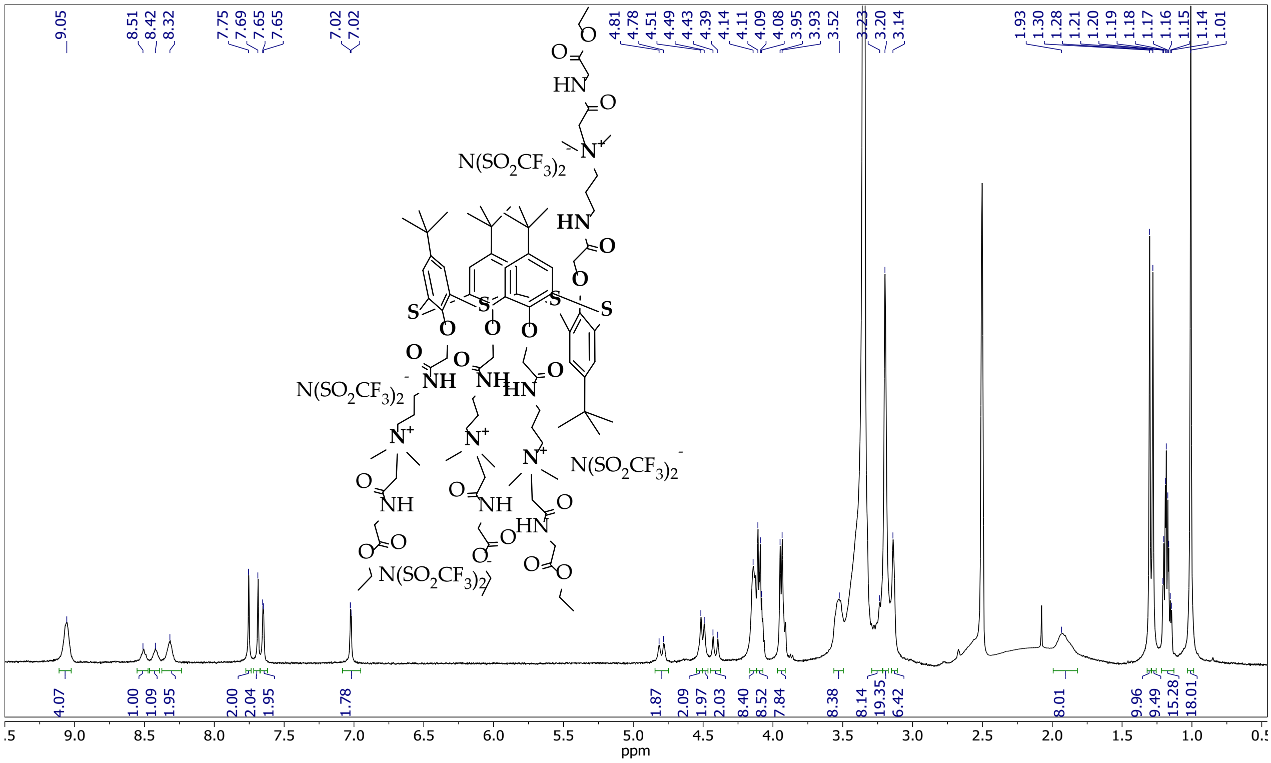

Figure S8. ^{1}H NMR spectrum of the compound **18** (*partial cone*), DMSO-d_6, 298 K, 400 MHz.

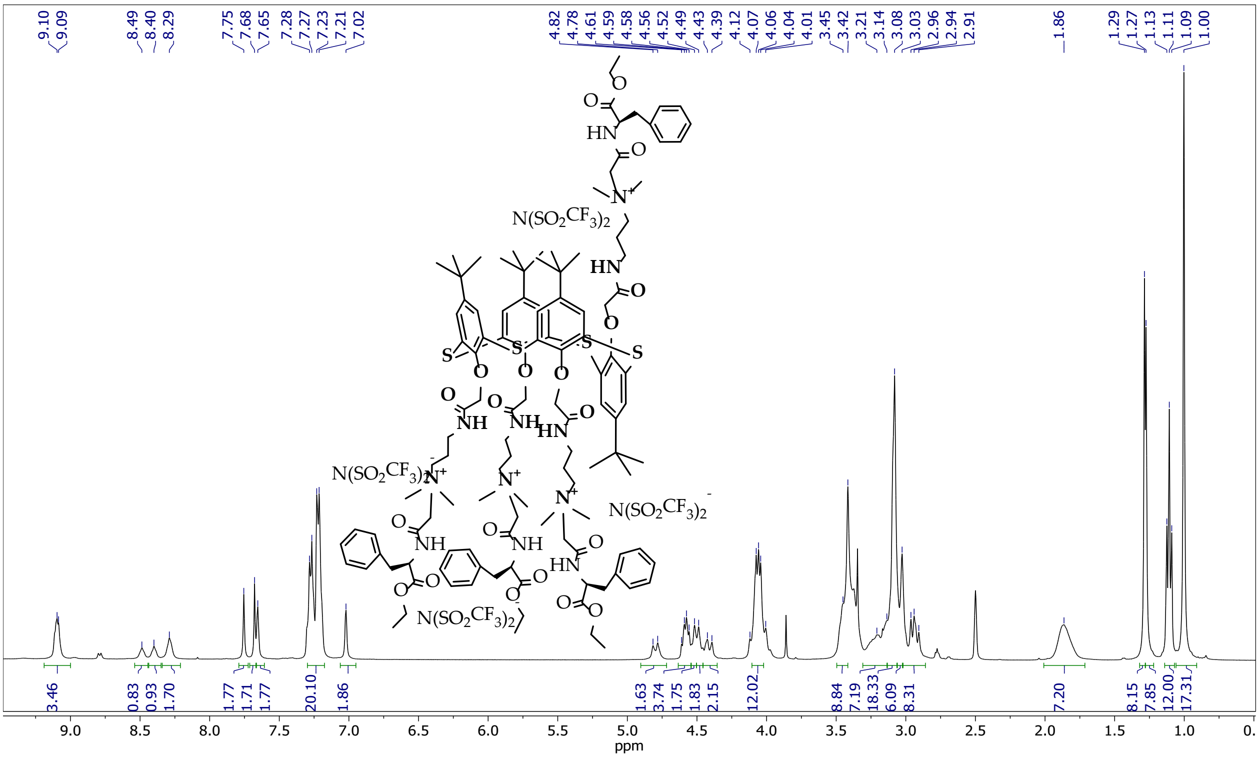

Figure S9. ^{1}H NMR spectrum of the compound **19** (*partial cone*), DMSO-d_6, 298 K, 400 MHz.

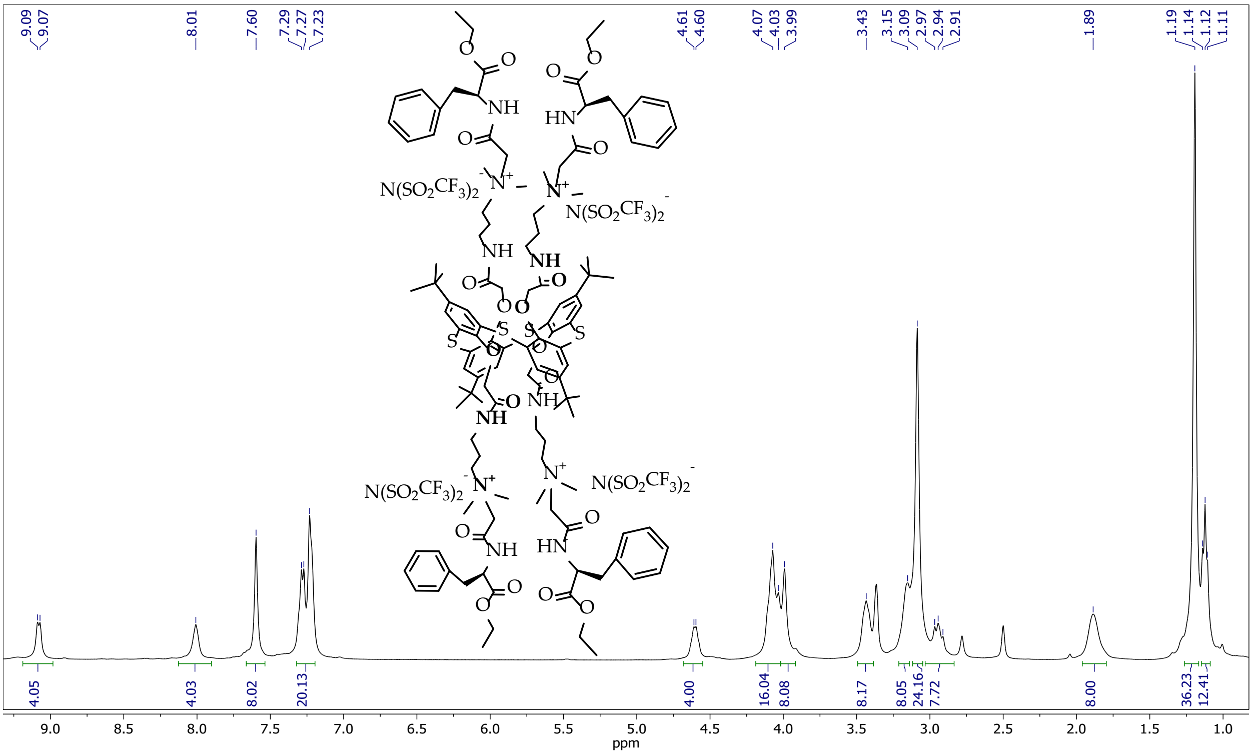

Figure S10. ^{1}H NMR spectrum of the compound **21** (*1,3-alternate*), DMSO-d_6, 298 K, 400 MHz.

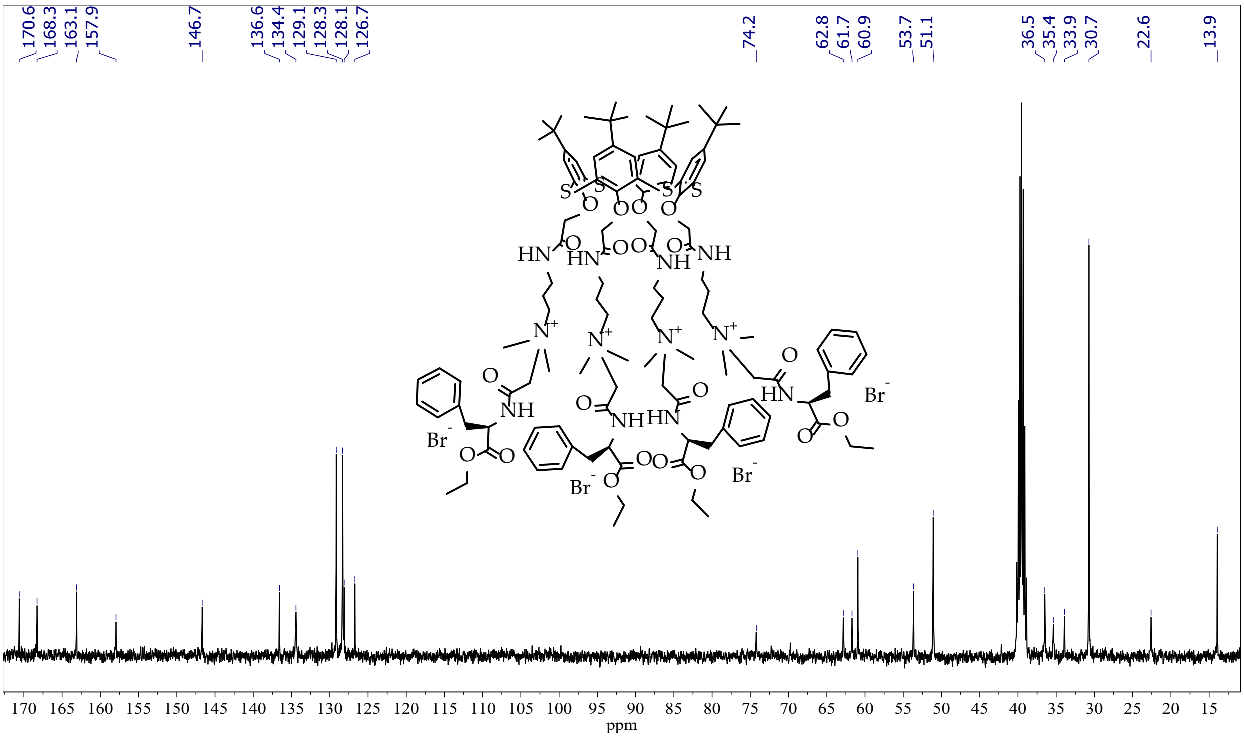

Figure S11. ^{13}C NMR spectrum of the compound **11** (*cone*), DMSO-d_6, 298 K, 100 MHz.

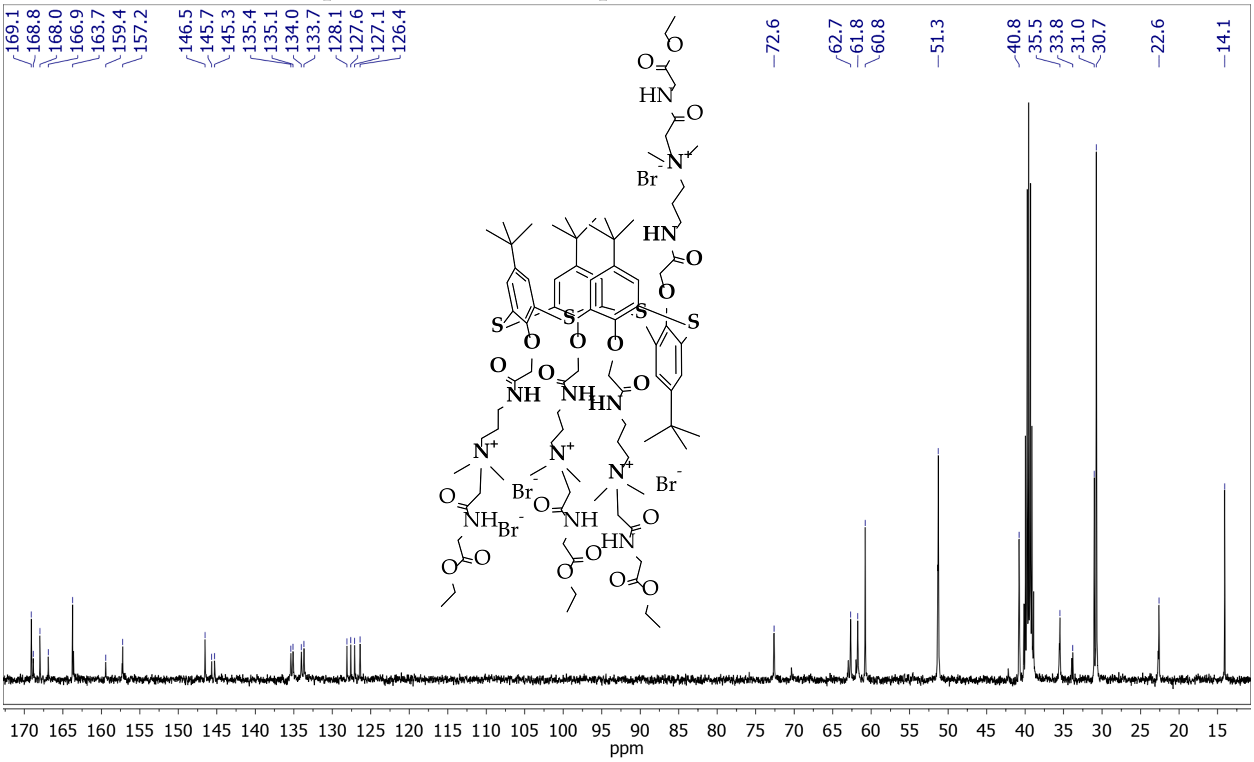

Figure S12. ^{13}C NMR spectrum of the compound **12** (*partial cone*), DMSO-d_6, 298 K, 100 MHz.

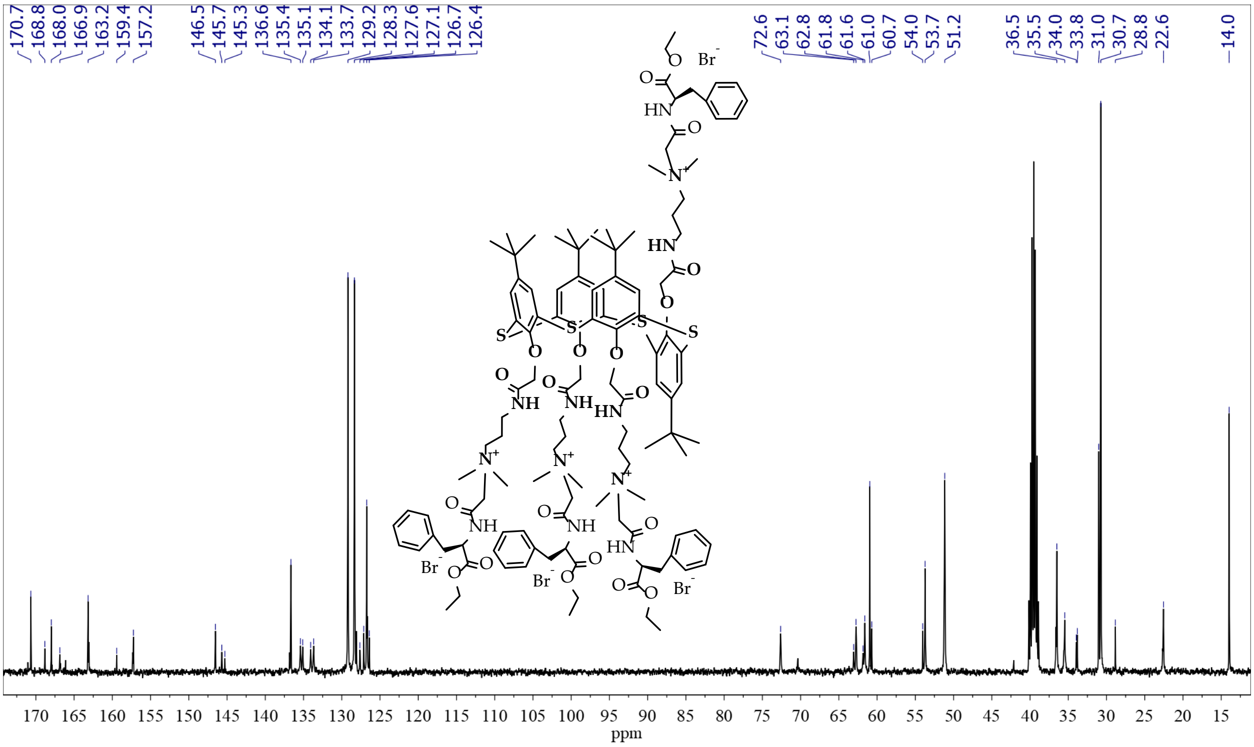

Figure S13. ^{13}C NMR spectrum of the compound **13** (*partial cone*), DMSO-d_6, 298 K, 100 MHz.

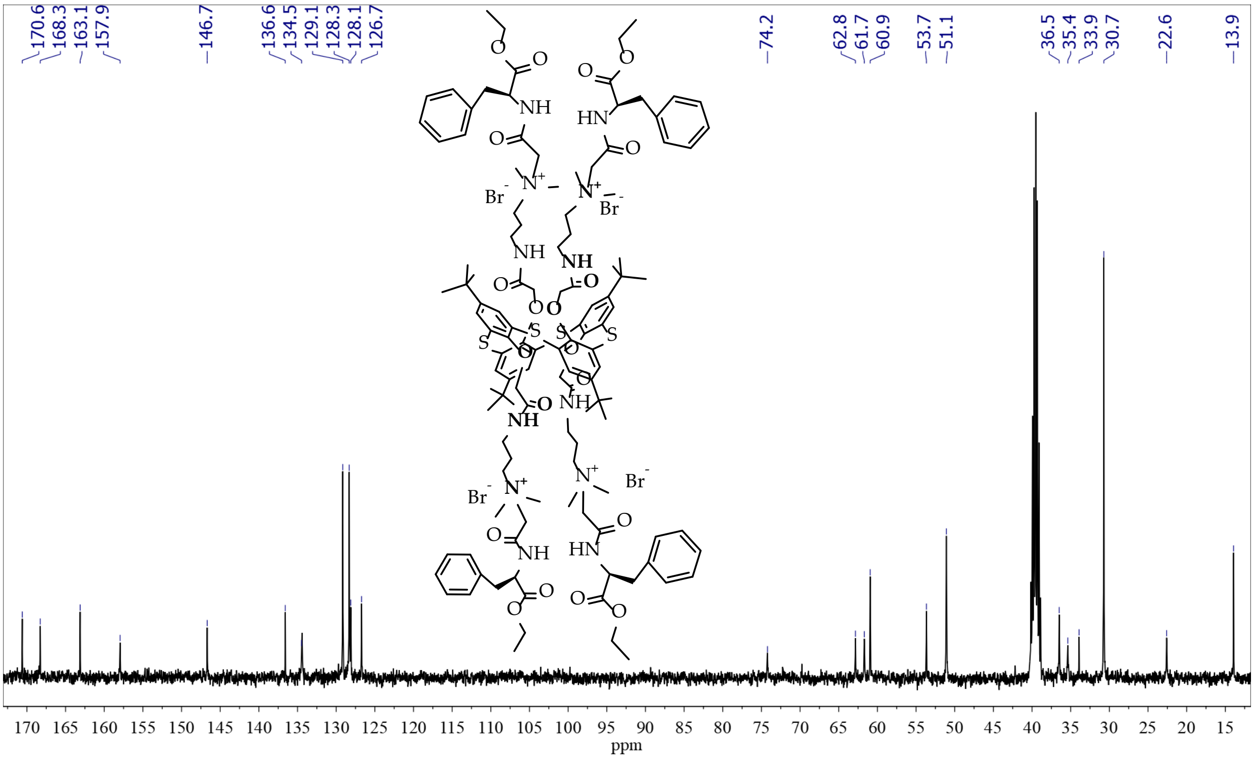

Figure S14. ^{13}C NMR spectrum of the compound **15** (*1,3-alternate*), DMSO-d_6, 298 K, 100 MHz.

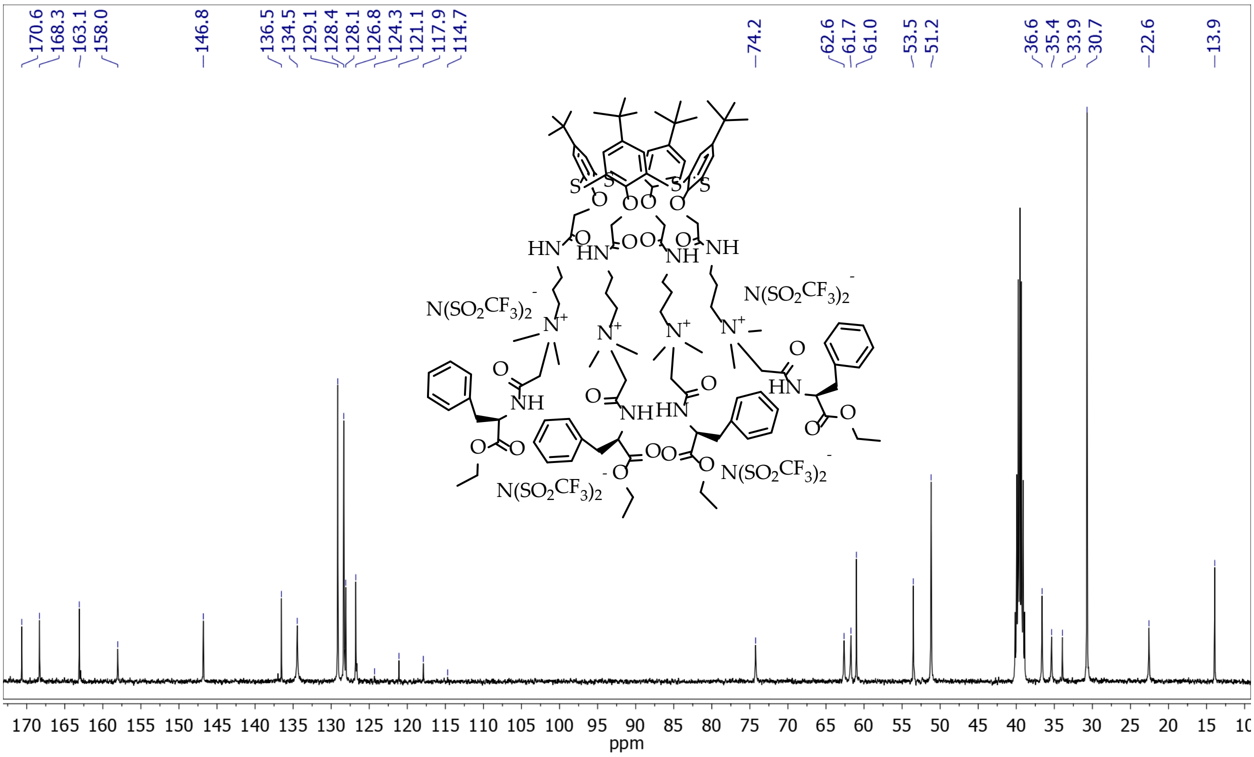

Figure S15. ^{13}C NMR spectrum of the compound **17** (*cone*), DMSO-d_6, 298 K, 100 MHz.

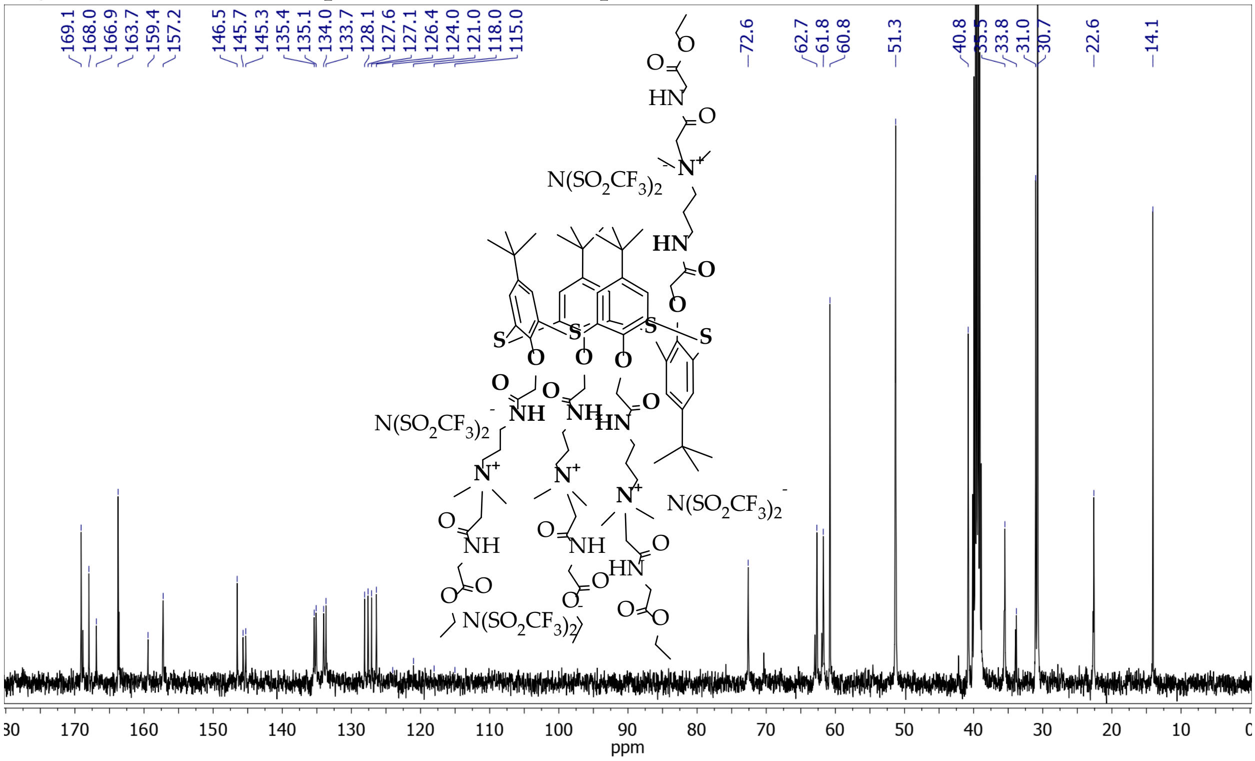

Figure S16. ^{13}C NMR spectrum of the compound **18** (*partial cone*), DMSO-d_6, 298 K, 100 MHz.

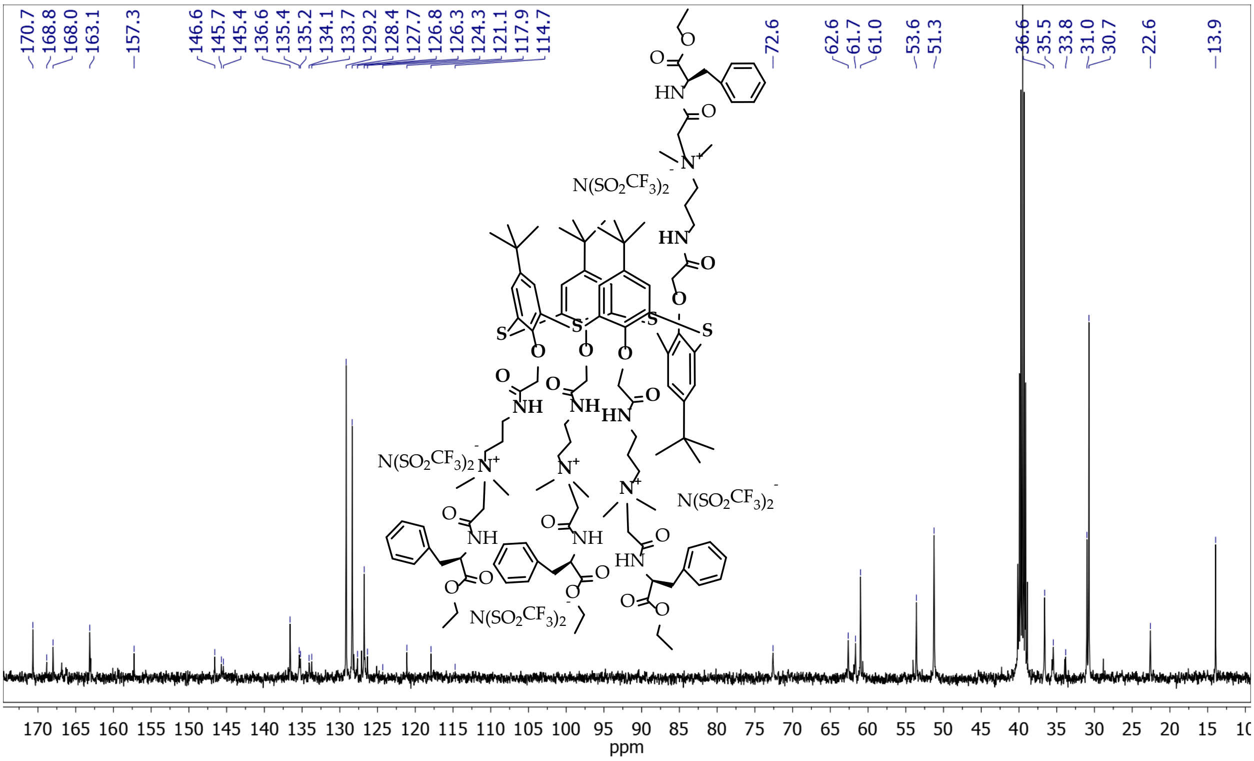

Figure S17. ^{13}C NMR spectrum of the compound **19** (*partial cone*), DMSO-d_6, 298 K, 100 MHz.

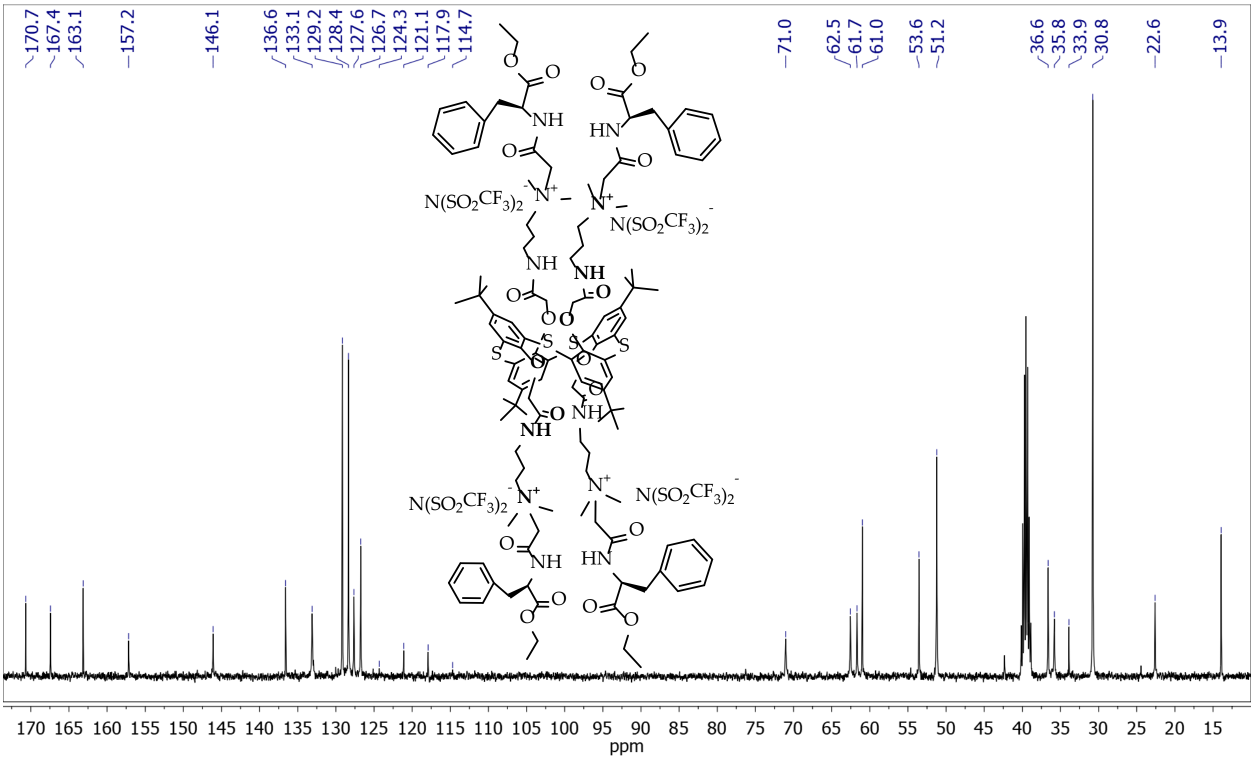

Figure S18. ^{13}C NMR spectrum of the compound **21** (*1,3-alternate*), DMSO-d_6, 298 K, 100 MHz.

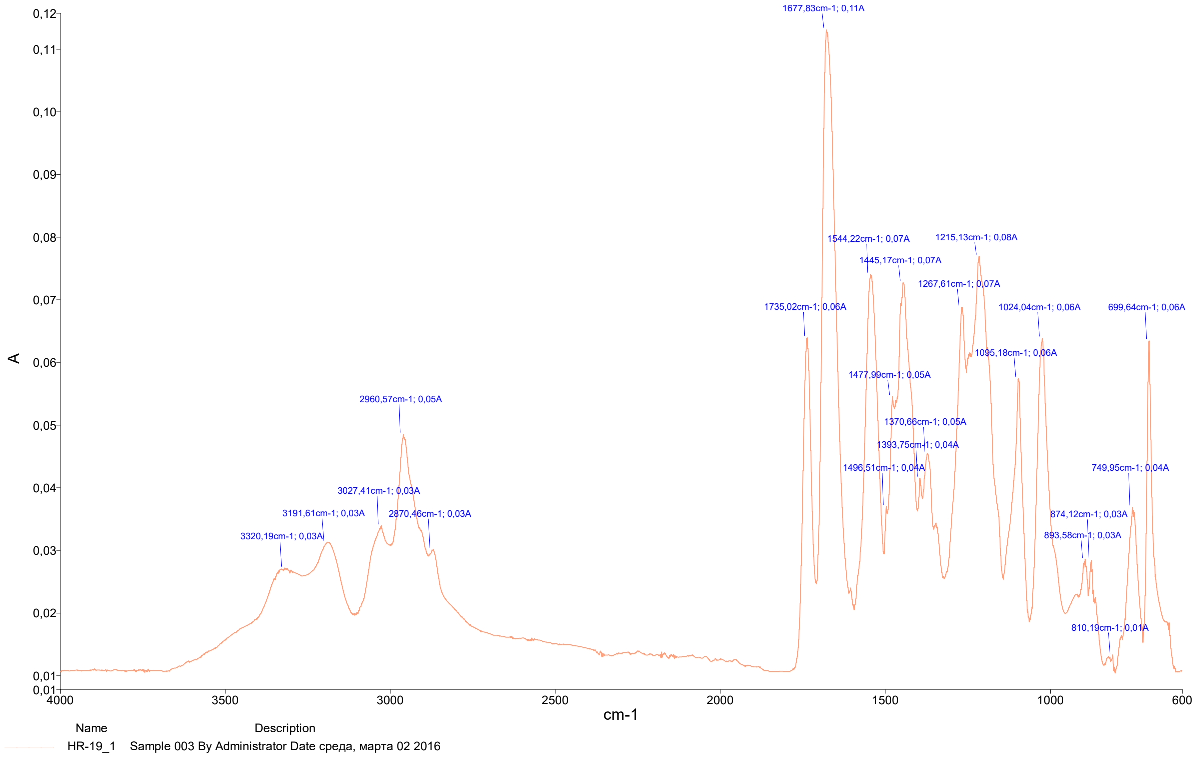

Figure S19. IR spectrum of the compound **11** (*cone*).

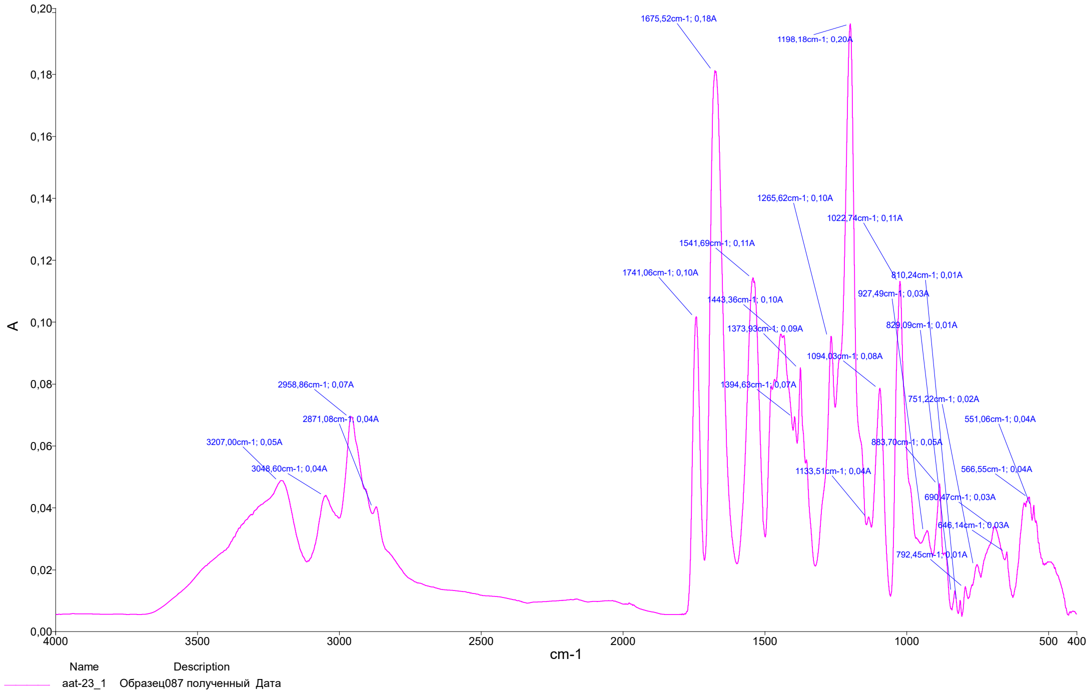

Figure S20. IR spectrum of the compound **12** (*partial cone*).

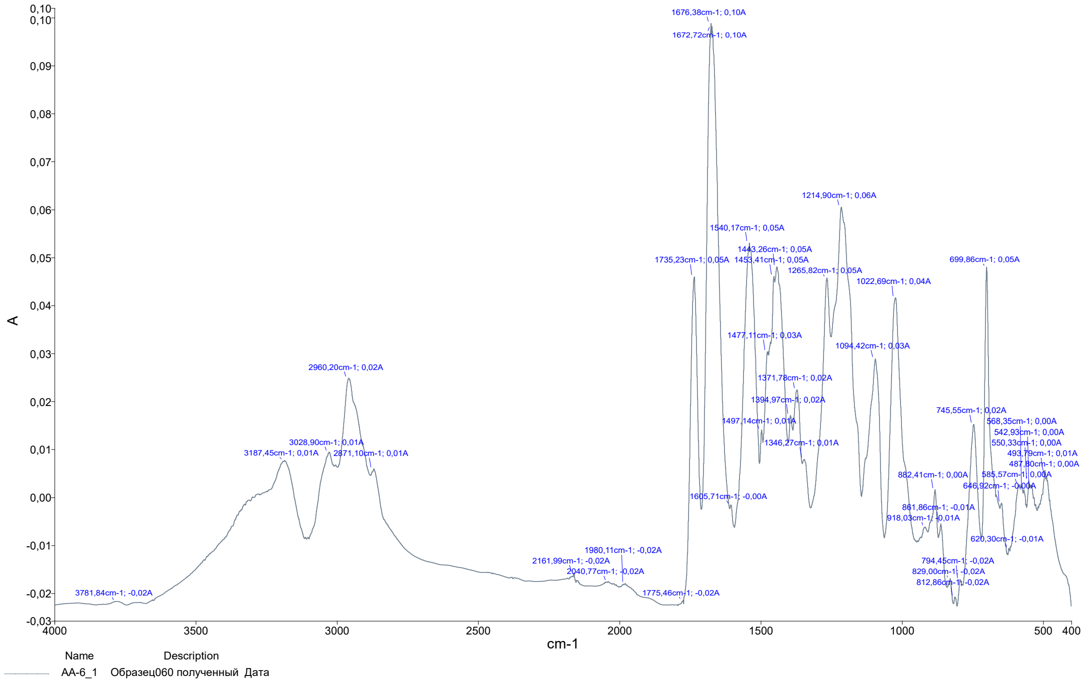

Figure S21. IR spectrum of the compound **13** (*partial cone*).

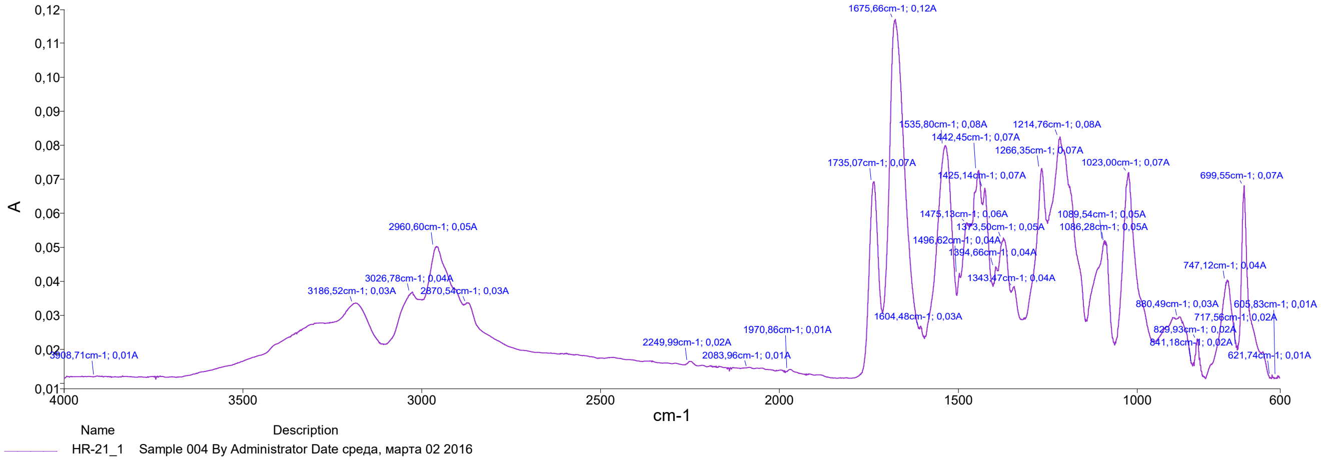

Figure S22. IR spectrum of the compound **15** (*1,3-alternate*).

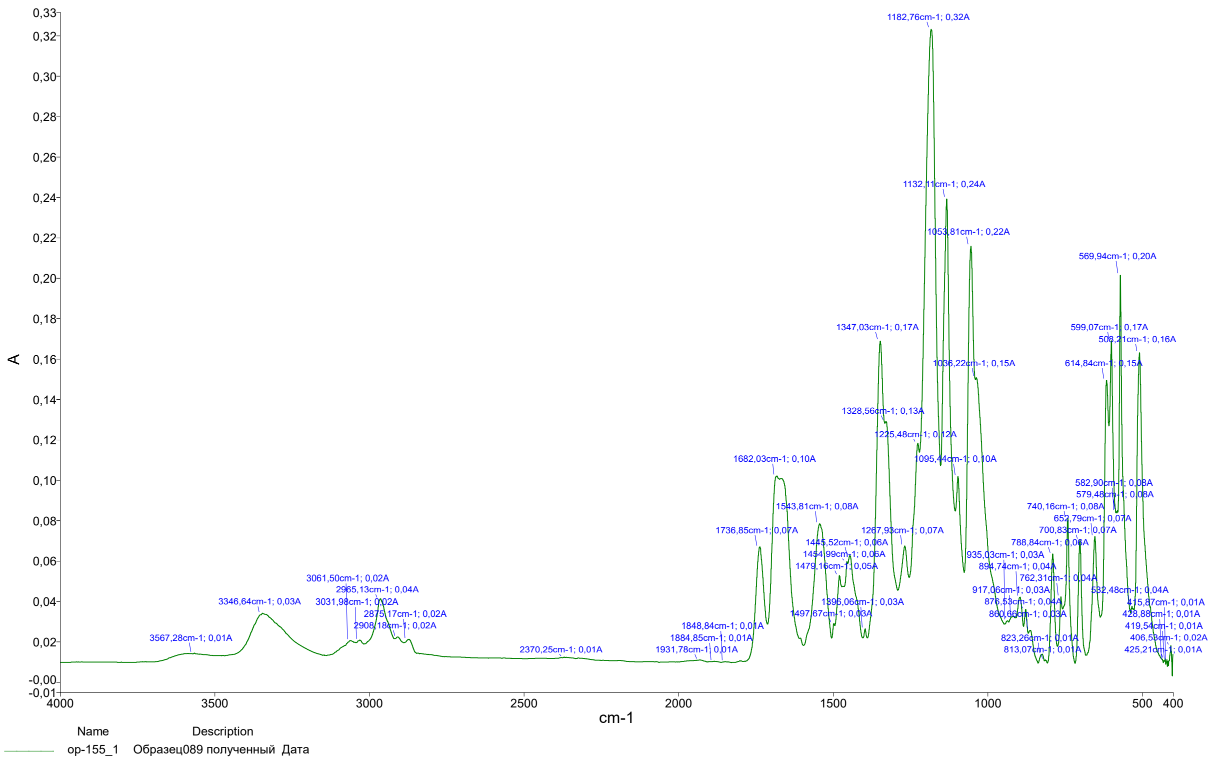

Figure S23. IR spectrum of the compound **17** (*cone*).

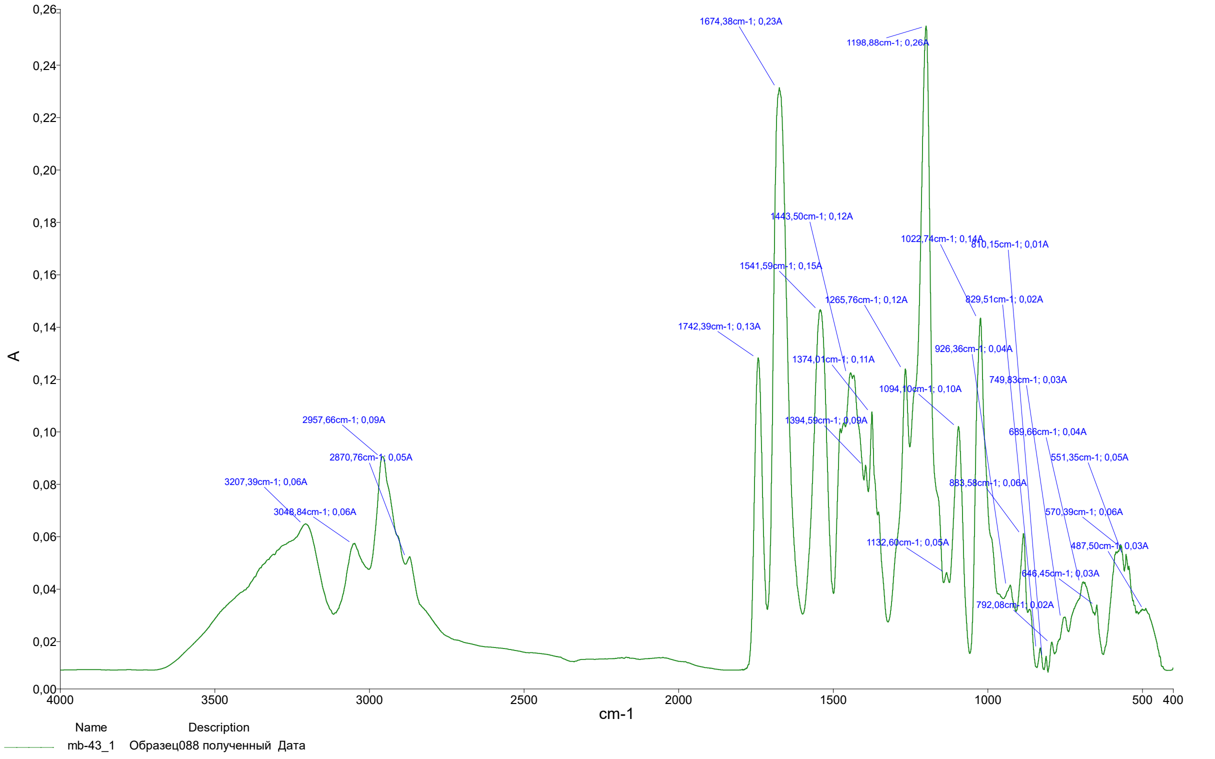

Figure S24. IR spectrum of the compound **18** (*partial cone*).

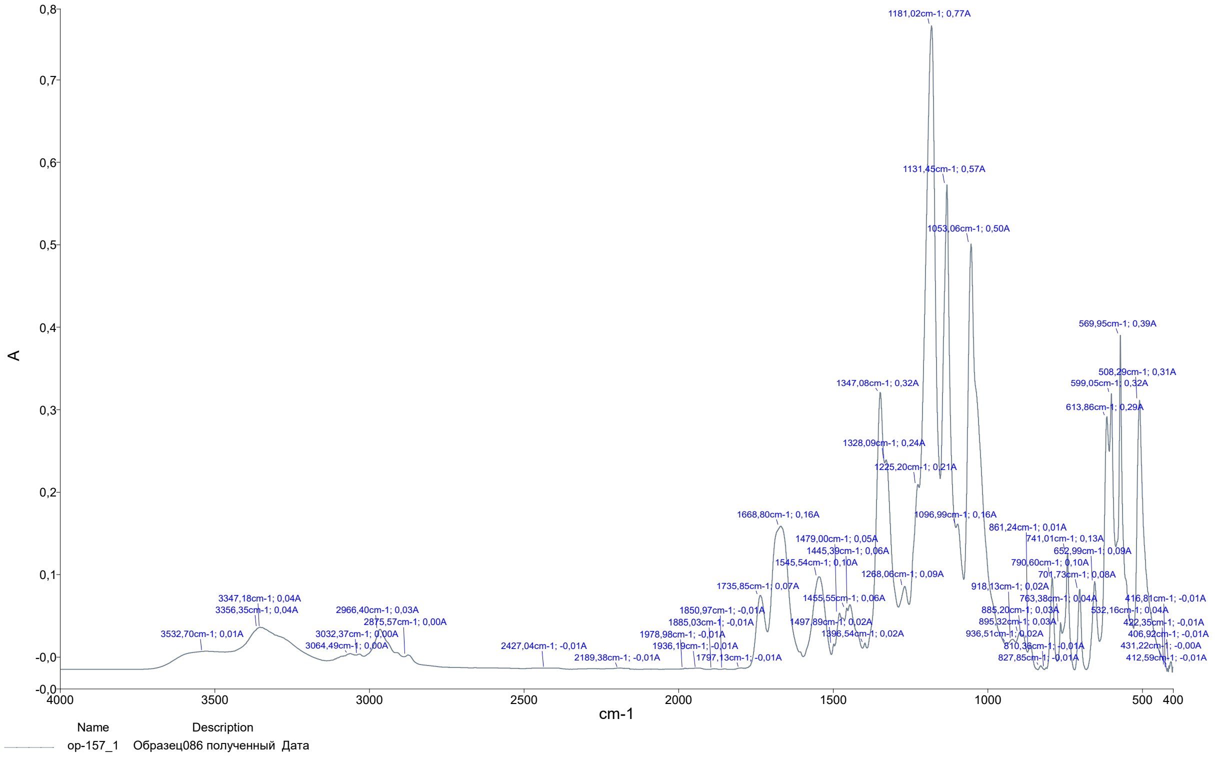

Figure S25. IR spectrum of the compound **19** (*partial cone*).

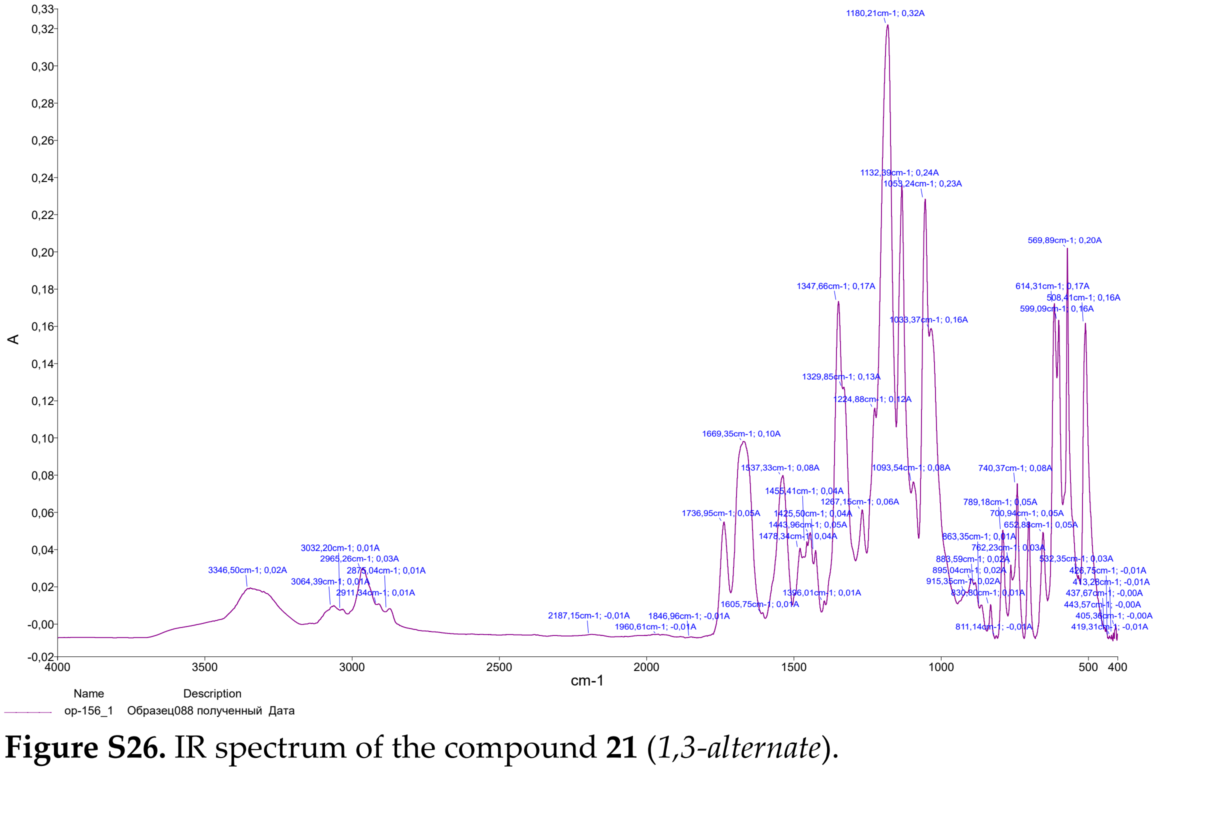

Figure S26. IR spectrum of the compound **21** (*1,3-alternate*).

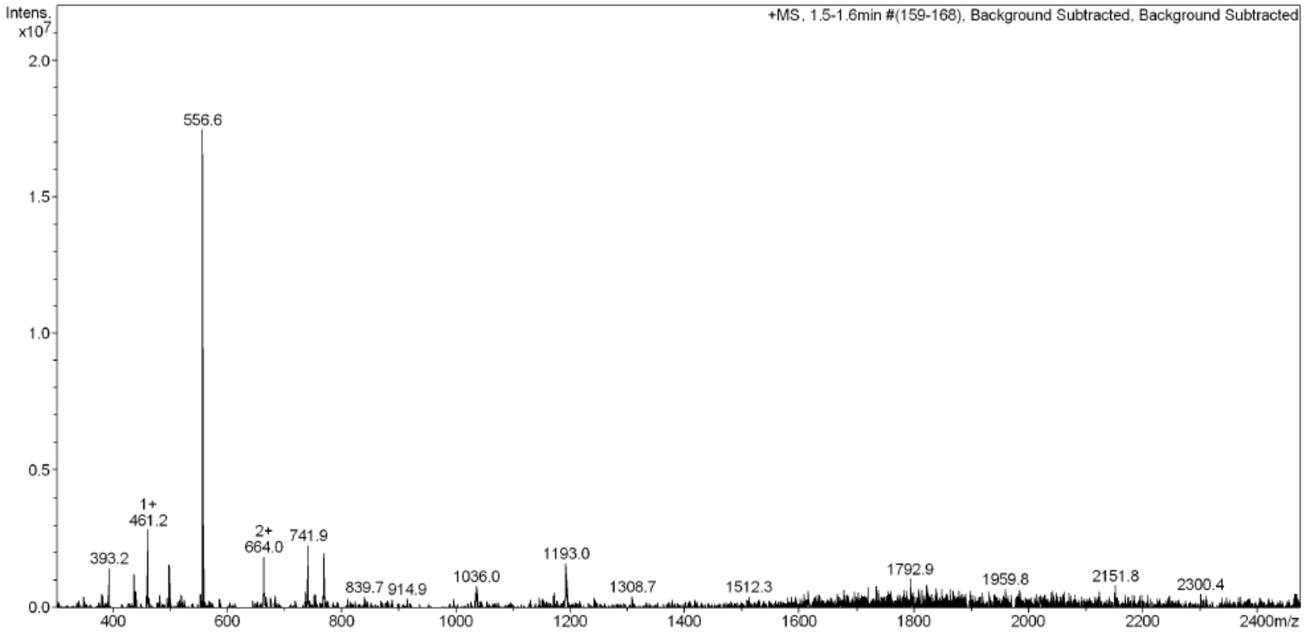

Figure S27. Mass spectrum (ESI) of the compound **11** (*cone*).

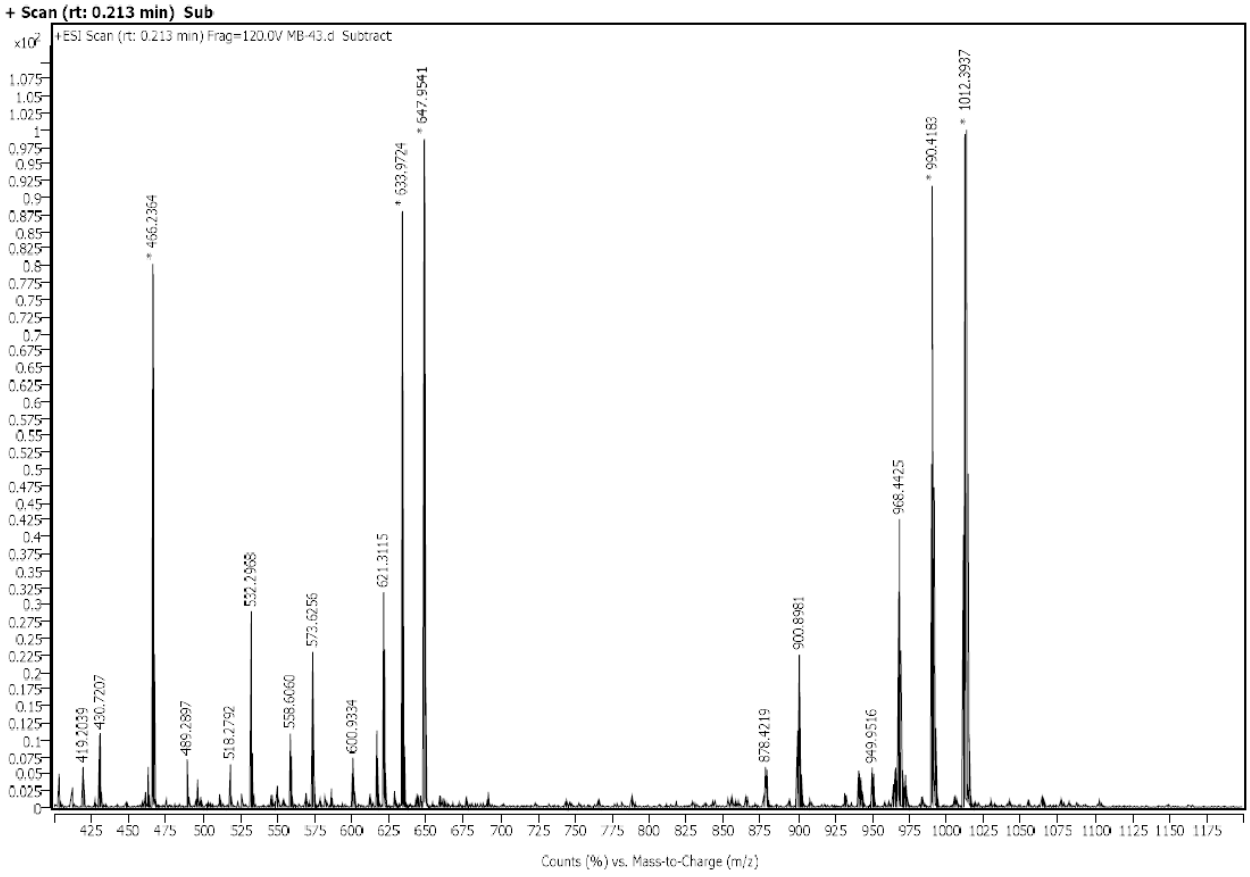

Figure S28. HR Mass spectrum (ESI) of the compound **12** (*partial cone*).

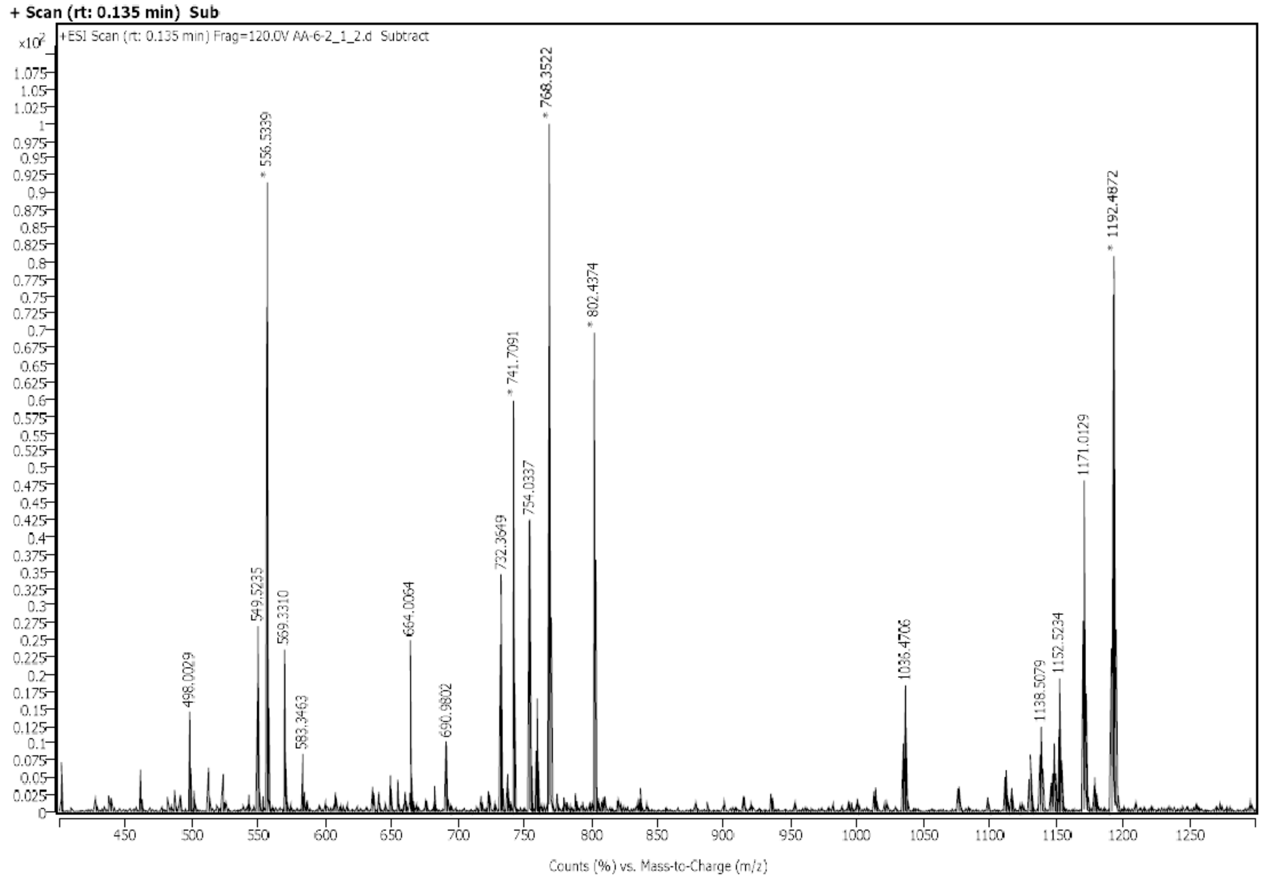

Figure S29. HR Mass spectrum (ESI) of the compound **13** (*partial cone*).

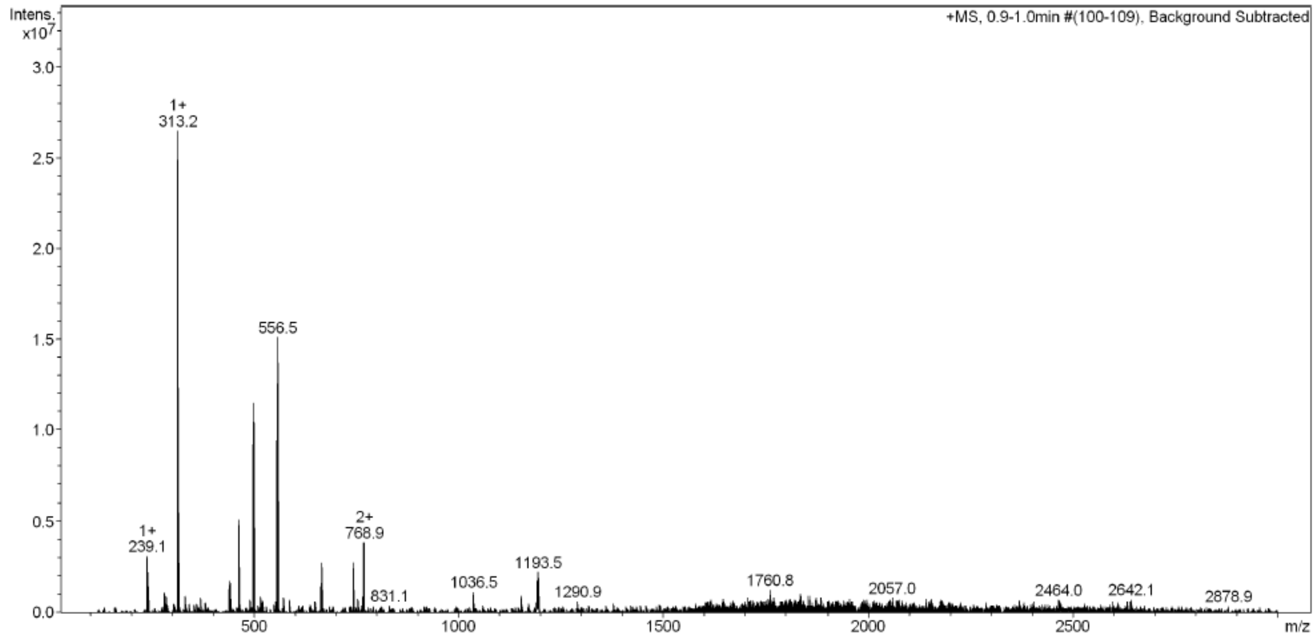

Figure S30a. Mass spectrum (ESI) of the compound **15** (*1,3-alternate*).

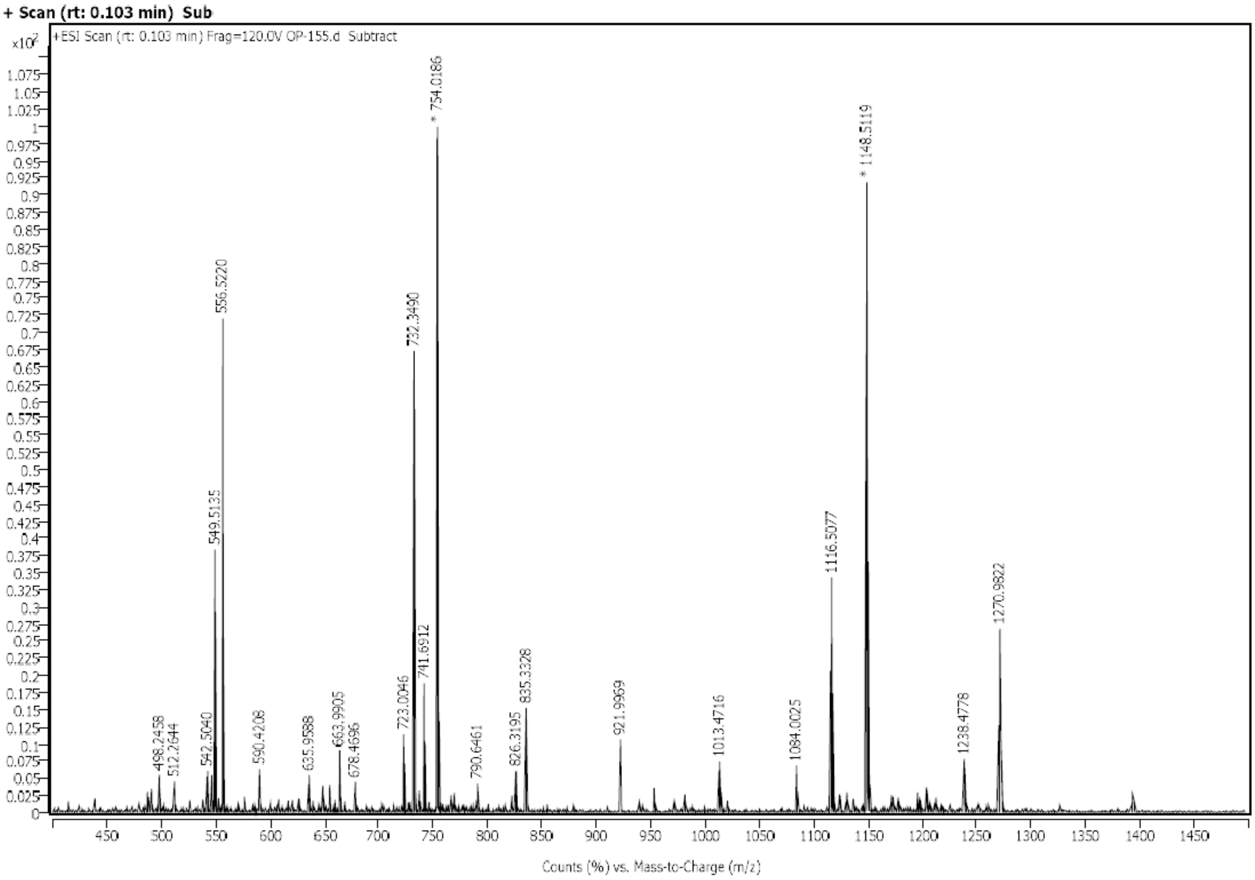

Figure S30b. HR Mass spectrum (ESI) of the compound **17** (*cone*).

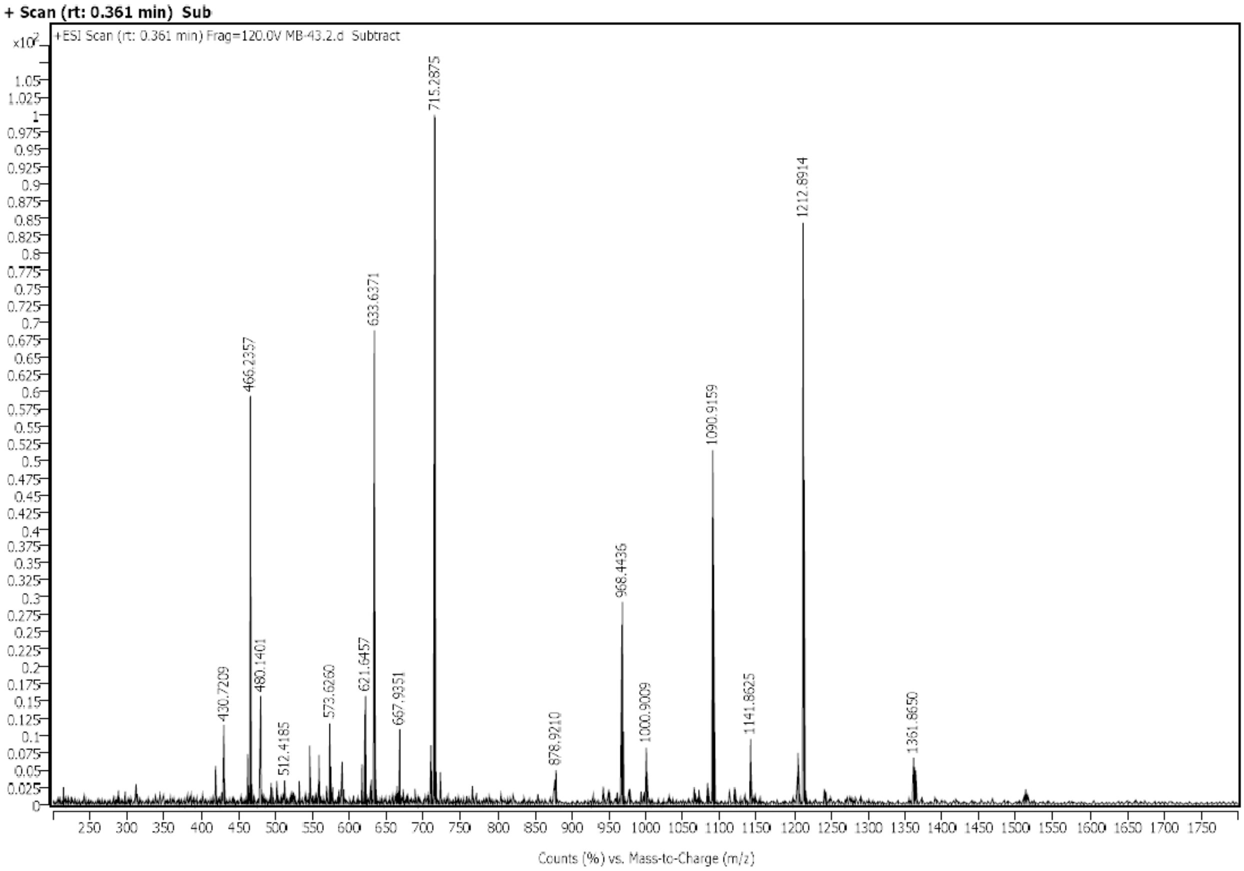

Figure S31. HR Mass spectrum (ESI) of the compound **18** (*partial cone*).

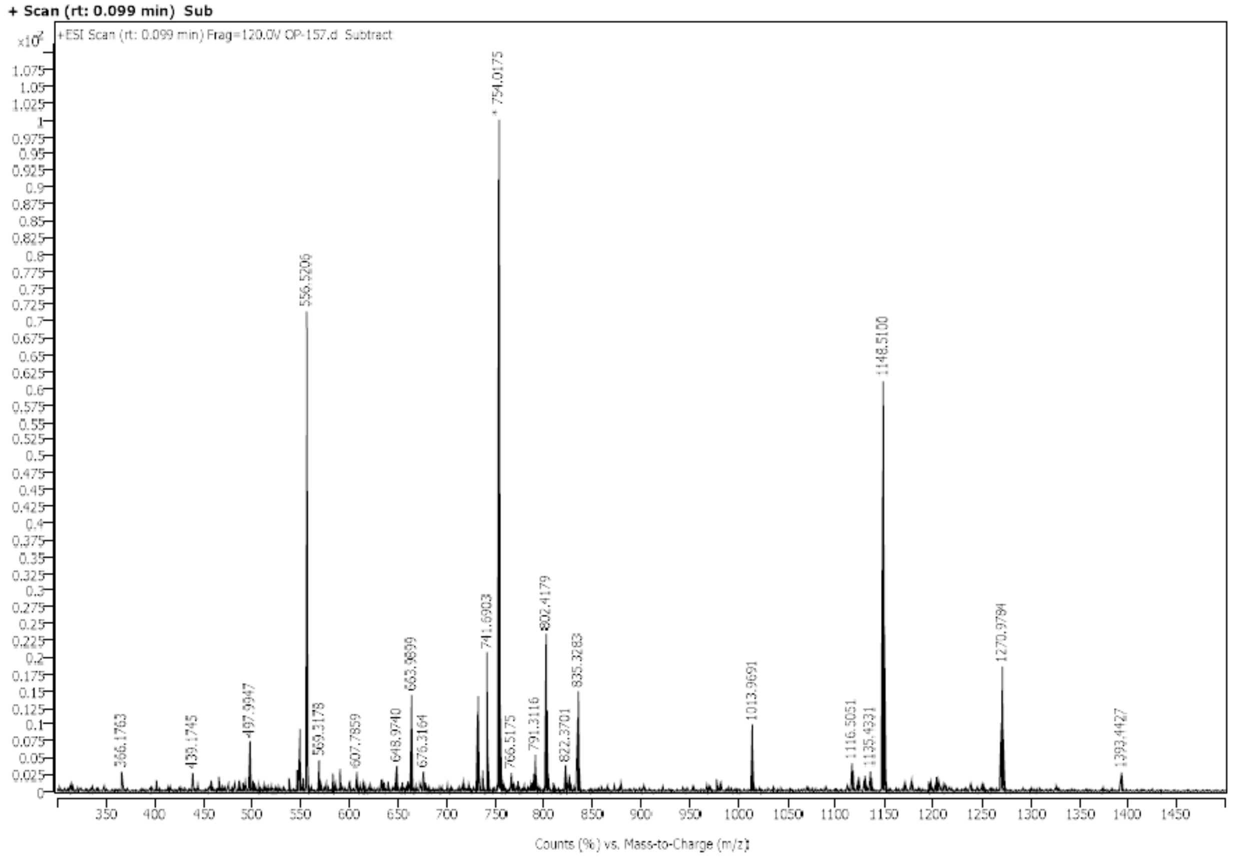

Figure S32. HR Mass spectrum (ESI) of the compound **19** (*partial cone*).

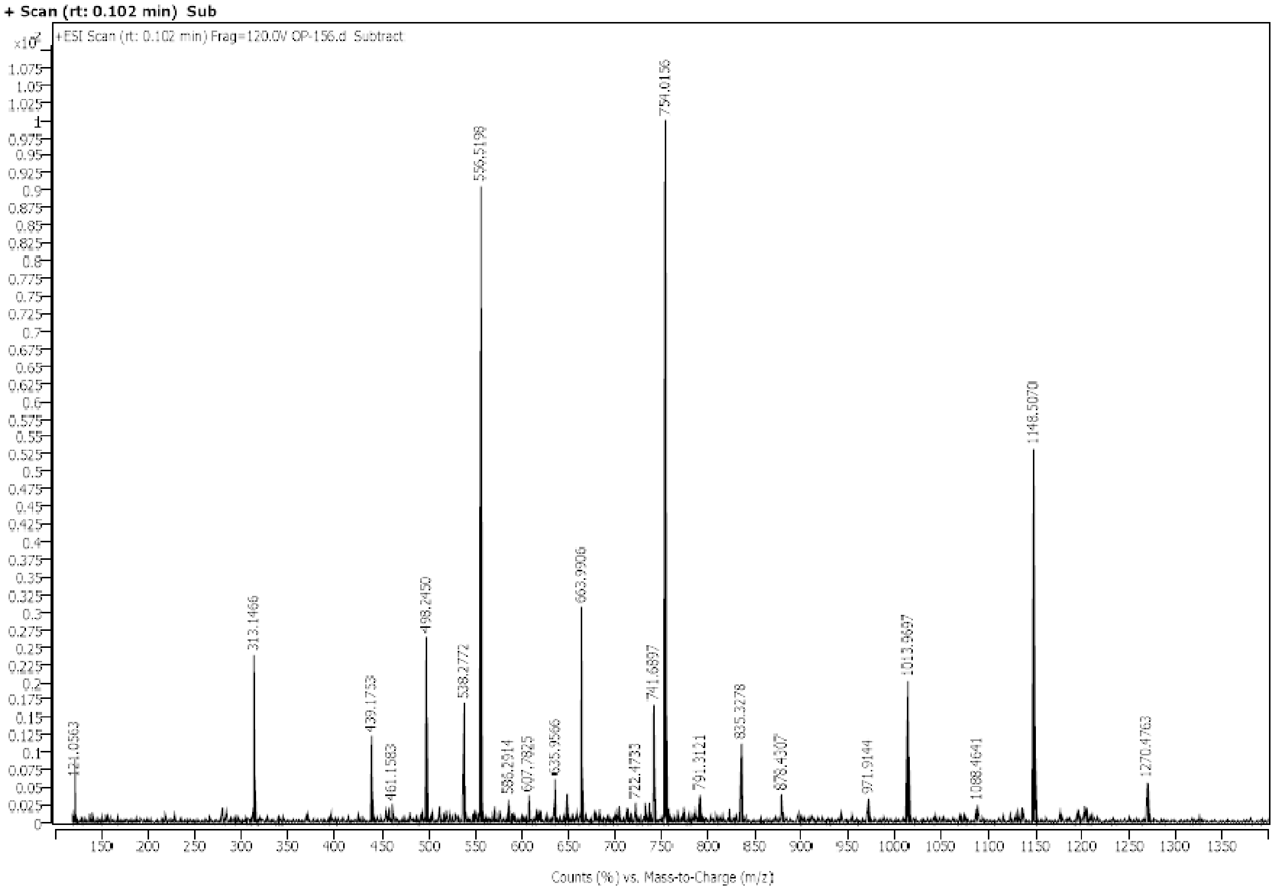

Figure S33. HR Mass spectrum (ESI) of the compound **21** (*1,3-alternate*).

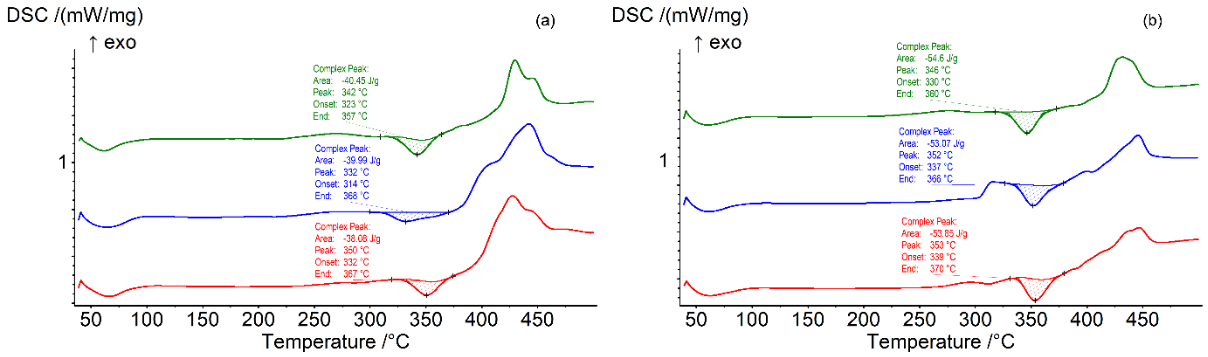

Figure S34. DSC curves of the compounds with Gly (a) **16** (green), **18** (blue), **20** (red), and *L*-Phe (b) **17** (green), **19** (blue), **21** (red) fragments (dynamic argon atmosphere of 75 ml/min in the temperature range from 40 to 500 °C).
